# T cell- but not tumor cell-produced TGF-β1 promotes the development of spontaneous mammary cancer

**DOI:** 10.18632/oncotarget.403

**Published:** 2011-12-31

**Authors:** Abira Sarkar, Moses K. Donkor, Ming O. Li

**Affiliations:** ^1^ Immunology Program, Memorial Sloan-Kettering Cancer Center, New York, NY 10065

**Keywords:** TGF-β, T cell tolerance, immunosurveillance, tumor immunity, metastasis, immunotherapy

## Abstract

During their development, tumors acquire multiple capabilities that enable them to proliferate, disseminate and evade immunosurveillance. A putative mechanism is through the production of the cytokine TGF-β1. We showed in our recent studies that T cell-produced TGF-β1 inhibits antitumor T cell responses to foster tumor growth raising the question of the precise function of TGF-β1 produced by tumor cells in tumor development. Here, using a transgenic model of mammary cancer, we report that deletion of TGF-β1 from tumor cells did not protect mice from tumor development. However, ablation of TGF-β1 from T cells significantly inhibited mammary tumor growth. Additionally, absence of TGF-β1 in T cells prevented tumors from advancing to higher pathological grades and further suppressed secondary tumor development in the lungs. These findings reveal T cells but not tumor cells as a critical source of TGF-β1 that promotes tumor development.

## INTRODUCTION

The biology that underlies tumor development is complex. Starting from tumor initiation, subsequent to cellular transformation, tumors undergo dramatic evolution[1]. During this multistep process, studies have demonstrated that tumors abandon most of the key features associated with normal cells and acquire capabilities that serve the ultimate goal of sustaining tumor cell survival and proliferation[1]. Indeed, the functional role of each of the hallmarks of cancer including proliferative signaling, evading growth suppressors, resisting death, enabling replicative immortality, inducing angiogenesis, and activating invasion and metastasis either directly or indirectly promotes tumor cell survival and/or proliferation[1]. While these hallmarks are intrinsic to tumor cells, growing evidence suggests that it is the totality of the tumor microenvironment that determines the outcome of carcinogenesis[1-3].

The immune system is postulated to recognize tumors and to protect the host from tumor development in a framework described as cancer immunosurveillance[4-6]. The immunosurveillance hypothesis envisaged that this function of effector leukocytes in defense against cancer would be necessary to maintain tissue homeostasis similar to its protective role against pathogenic microbes. Studies in mice have demonstrated that deficiency in recombination-activating gene (*Rag*)*-1* or *-2* required for the development of adaptive immunity results in increased frequency, shortened tumor latency, faster tumor growth kinetics of chemical-induced sarcomas and increased development of a broader spectrum of spontaneous tumors compared to age-matched wild type littermates[6-8]. Tumors that develop under immunodeficient *Rag-2* knockout conditions are rejected when transplanted into wild type recipients but develop in hosts depleted of CD4^+^ or CD8^+^ T cells[7, 9]. Moreover, tumors derived from wild type mice are not rejected when transferred into either wild type mice or *Rag-2*-deficient hosts[7, 9]. These findings support the immunosurveillance hypothesis and further demonstrate that T cells not only mediate tumor elimination but can also shape the immunogenicity of tumors.

Recently, some effector mechanisms that mediate lymphocyte-dependent tumor rejection have been defined. Modification of tumor cells to secrete interferon (IFN)-γ resulted in the induction of persistent specific T cell immunity and a CD8^+^ T cell-dependent tumor rejection whereas treatment of wild type mice with antibodies to neutralize endogenous IFN-γ led to failed rejection of carcinogen-induced tumors[10-12]. In addition to IFN-γ, the cytotoxic granule pathway has been shown to be essential for immunosurveillance. Deficiency in perforin, a crucial pore-forming protein required for the killer function of CTLs and natural killer cells results in development of fibrosarcomas with reduced latency and greater incidence and the mice further show greater susceptibility to the development of B cell lymphomas in comparison to control wild type mice[13-16]. These findings collectively underscore type 1-lymphocyte responses in the immunosurveillance of spontaneous and MCA-induced tumors.

However, for the vast majority of tumor types, immune-mediated tumor rejection is not observed. In a transgenic model of sporadic cancer, the well-characterized SV40 Tag oncogene is stochastically activated leading to tumor development in diverse unpredictable tissues[17]. In this model, CD8^+^ T cells recognize the Tag tumor-associated antigen but fail to kill target cells when tested *in vivo*[17]. In the TRAMP (transgenic adenocarcinoma of mouse prostate) model of prostate cancer, our recent findings, together with previous observations, demonstrated that HRC CD8^+^ T cells specific for the TRAMP self-antigen histone H4 show functional defects [18, 19]. CD8^+^ T cells specific to tumor antigens have also been described in a subset of cancer patients but similar to the animal models, these T cells show limited effector activity[20, 21]. These findings collectively suggest that T cells are able to recognize their cognate tumor antigens but the tumors avoid destruction by inducing T cell tolerance.

How tumors initially prime T cell recognition and subsequently escape T cell-mediated eradication remains poorly understood. Understanding the mechanistic underpinnings of T cell tolerance in tumors is fundamental to the development of more effective anticancer therapies. It is conceivable that the six hallmarks of cancer that functionally converge on tumor cell survival and chronic proliferation can each influence antitumor immune response. Indeed, the acquisition of each succeeding hallmark capability likely represents an evolutionary step whose success would require some escape from immune barrier to tumor progression. In addition, studies have shown that other cell-biologic behaviors of tumors do not favor the generation or function of effector T cells. For example, tumors are frequently associated with downregulation of MHC class I, impaired antigen presentation, lack of costimulation, exapansion and activation of suppressor cells which results in defective T cell responses to tumors [2, 22, 23].

The tumor microenvironment is a dynamic complex milieu consisting of the tumor, stromal cells such as fibroblasts and immune cells, vascular and lymphatic networks and the extracellular matrix. Beside providing a supportive stroma, studies have revealed that host cells can also influence tumor phenotype by supplying a variety of bioactive agents such as growth factors, cytokines and chemokines that can modulate tumor growth[1-3, 24]. It is possible that tumor progression requires these secreted factors although given the growth autonomy that malignancies acquire during their evolution it remains unclear as to what extent tumors depend on these factors for their growth. Recent studies have shown that the function of these secreted factors in tumors is in large part the inhibition of antitumor immune responses [1-3, 24].

The identity, sources and mechanisms of how some of these secreted factors regulate T cell responses to tumors have begun to be understood. In the stochastic model of cancer, Willimsky and Blankenstein showed that the development of defective tumor-specific T cell responses is coincident with loss of IFN-γ response and the acquisition of elevated levels of the cytokine transforming growth factor (TGF)-β1 in the circulation[25]. Systemic levels of TGF-β1 are also associated with T cell dysfunction in cancer patients where it is a negative prognostic indicator[26]. TGF-β is a family of three isoforms comprising TGF-β1, 2 and 3. It is secreted as an inactive latent ligand that requires activation catalyzed by a variety of factors including proteases and integrins [27-31]. Once the active ligand is liberated, it binds the serine or threonine kinases TGF-β type I (TGF-βRI) and type II (TGF-βRII) receptors, which recruit and phosphorylate Smad2 and Smad3 transcription factors[28, 29, 32]. Activated Smad2 and Smad3 proteins dimerize and complex with Smad4 and translocate to the nucleus to regulate Smad-dependent genes[28, 29, 32]. Studies have shown that TGF-β exerts antiproliferative effects on epithelial cells by transcriptional repression of c-Myc and induction of inhibitors of cyclin-dependent kinases, which are important for cell cycle entry and progression[29]. In addition, TGF-β profoundly represses lymphocyte proliferation and differentiation and autoreactive function[28, 32-37].

In a variety of tumor types, TGF-β has been suggested to play an important role in tumor progression[38]. At the early stage of tumor development, TGF-β is thought to regulate cellular homeostasis by enforcing cytostasis, differentiation and apoptosis of tumor cells[39, 40]. Indeed, transgenic expression of a dominant negative mutant of TGF-βRII (DNR) in prostate epithelium accelerates tumor development and reduces tumor latency in TRAMP mice, underscoring a suppressive function of TGF-β in tumorigenesis[41]. However, studies show that some tumors can circumvent the suppressive effects of TGF-β via mutations in TGF-β receptors or downstream alterations that disable the tumor suppressor arm of the TGF-β pathway[42, 43]. Autocrine TGF-β signaling is then thought to promote tumor progression via regulation of the invasion-metastasis cascade involving epithelial-to-mesenchymal transition, invasion of regional tissue, dissemination and metastatic colonization [29].

In contrast to this prevailing model that emphasizes the effect of TGF-β signaling on tumors, recent studies suggest that TGF-β regulation of T cell responses to tumors is an integral part of tumor development[18, 44-47]. Consistent with these observations, we recently demonstrated that failure to control TRAMP tumor growth by adaptive immunity arises from T cell-intrinsic TGF-β signaling that initiates at the tumor-draining lymph nodes[18]. TRAMP tumors, like most tumors, are sensitive to TGF-β ligands but the requirement of TGF-β signaling for the development of these tumors remains to be determined. Nonetheless, these observations suggest that T cell-intrinsic TGF-β signaling probably exercises greater control on tumor development in comparison to tumor responsiveness to TGF-β. Importantly, it can be inferred from these findings that the varied tumor immunoevasion strategies and the cell-intrinsic hallmarks of cancer can be overruled by blocking TGF-β signaling in T cells.

Diverse cell types in normal tissues produce TGF-β1 and its production may further be upregulated in response to tumor development [34, 48-50]. There is, however, a strong sense that tumor secretion of TGF-β1 is a major mechanism of T cell tolerance [44, 51-53]. It has been shown that overexpression of an active form TGF-β1 in an immunogenic tumor cell results in inhibition of CTL-mediated tumor rejection when inoculated into mice[53]. On the basis of this overexpression study and other succeeding studies, it was concluded that tumors produce TGF-β1 to evade immune control[44, 51, 52]. Tumor secretion of TGF-β1 is therefore conceptualized to dampen T cell reactivity at the tumor microenvironment where T cells are exposed to large quantities of TGF-β1 produced by tumor cells. Contrary to this view, we demonstrated in our recent studies that T cell-specific deletion of TGF-β1 protected mice against tumor growth and metastasis independent of TGF-β1 produced by tumor cells[18]. In *Tgfb1*^f/n^ *Cd4cre*-TRAMP mice, we found that protective tumor immunity was associated with T cell differentiation into IFN-γ- and granzyme (Gzm)B-producing effectors in the tumor draining lymph nodes and prostate[18] in line with reports showing that TGF-β1 suppressess antitumor immunity by transcriptional repression of the CTL program including the expression of IFN-γ and GzmB[54]. These studies provide compelling evidence that T cell TGF-β1 fosters T cell tolerance in tumors and is essential for tumor growth. Importantly, these observations do not support an essential role for TGF-β1 produced by tumor cells in the development of these tumors. Nevertheless, in the absence of experimental approaches that assess the definitive function of tumor-produced TGF-β1, evidence against tumors as the critical source of TGF-β1 that inhibits immunosurveillance would be incomplete.

In this study, we addressed the specific function of tumor-produced TGF-β1 in tumor development by crossing mice carrying floxed/null alleles of *Tgfb1* to the *Mmtvcre* deleter mouse strain in which cre recombinase directs deletion of the *Tgfb1* allele from mammary epithelium[55]. The *Tgfb1*^f/n^ *Mmtvcre* line and the *Tgfb1*^f/n^ *Cd4cre* line used in our previous studies[18] were further crossed to the polioma middle T antigen (PyMT) mice in which the transgenic mice develop highly aggressive mammary adenocarcinoma that metastasizes to the lungs[56]. We found that tumor growth and pulmonary metastasis were comparable between *Tgfb1*^f/n^ *Mmtvcre*-PyMT and control *Tgfb1*^f/n^-PyMT littermates. However, deletion of TGF-β1 from T cells inhibited tumor development and protected PyMT mice from pulmonary metastasis. These findings establish that TGF-β1 produced by T cells, but not tumor cells, is required for tumor growth and metastasis in PyMT mice. These observations further extend the generality of the requirement of TGF-β1 produced by T cells in the development of spontaneous tumors. ResultsDeletion of tumor-produced TGF-β1 does not affect tumor growth and metastasis in PyMT mice

**Figure 1:**
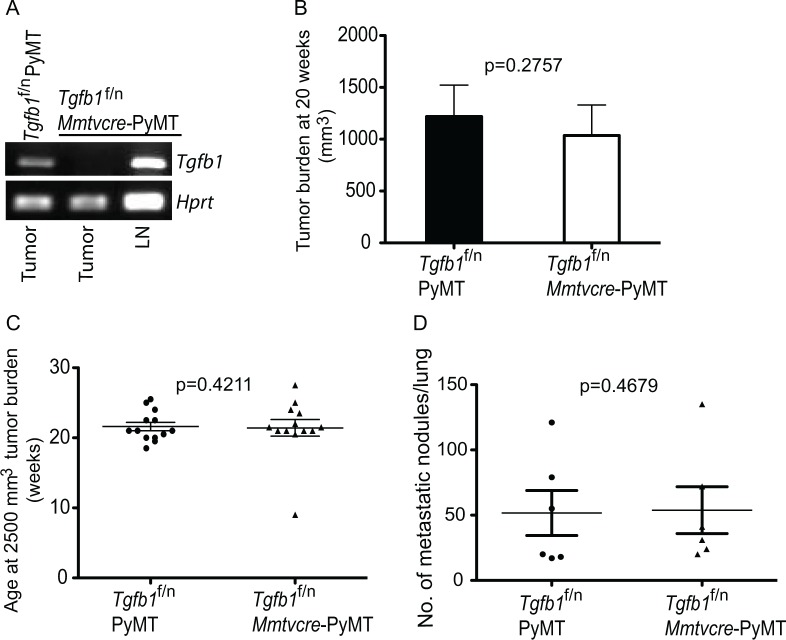
Absence of TGF-β1 produced by mammary tumor cells does not affect tumor growth (A) RT-PCR analysis of *Tgfβ1* and *Hprt* expression from FACS-sorted tumor cells of control *Tgfb1*^f/n^ PYMT mice, and from tumor cells and lymph node cells of *Tgfb1*^f/n^ *Mmtvcre*-PYMT mice. LN; lymph node (B) Total tumor burden in all mammary glands of *Tgfb1*^f/n^ *Mmtvcre*-PYMT (n=9) and control Tgfb1^f/n^ PYMT (n=9) mice at 20 weeks of age. Tumor volume was calculated using the formula L x W^2^x 0.52 (where L is the longest diameter and W is the smallest diameter.) (C) The age at which *Tgfb1*^f/n^ *Mmtvcre*-PYMT (n=9) and littermate control *Tgfb1*^f/n^ PYMT mice (n=9) achieved the predetermined tumor burden of 2500 mm^3^. (D) Total pulmonary metastatic nodules in *Tgfb1*^f/n^ *Mmtvcre*-PYMT (n=5) and control *Tgfb1*^f/n^ PYMT (n=6) mice were assessed at a tumor burden of 2500 mm^3^. The p values between the two groups of tumor burden (B) age (C) and number of metastatic nodules (D) are shown (Students t-test).

In addition to the cell intrinsic hallmarks of cancer, tumors require other enabling mechanisms to achieve their survival and chronic replication program[1]. Studies show that the ability to evade immune eradication via TGF-β-mediated tolerance induction is a crucial survival mechanism for tumors[1, 2, 39, 48, 57, 58]. Recently, we demonstrated a non-redundant function of TGF-β1 produced by T cells in tumor development [18], which leaves open the exact function of TGF-β1 produced by tumors in tumor development. To rigorously test the requirement of tumor TGF-β1 for the development of spontaneous tumors, we employed genetic approach targeting TGF-β1 produced by tumor cells. To this end, we bred mice carrying floxed/null alleles of *Tgfb1* (*Tgfb1*^f/n^) mice to the *Mmtvcre* deleter strain in which the cre recombinase instructs allelic recombination in mouse mammary epithelium[55]. *Tgfb1*^f/n^ *Mmtvcre* mice were further crossed to PyMT mice to obtain *Tgfb1*^f/n^ *Mmtvcre*-PyMT mice. By RT-PCR analysis, we verified that TGF-β1 is specifically deleted in tumor cells but not lymph node cells of tumor-bearing *Tgfb1*^f/n^ *Mmtvcre*-PyMT mice (Figure 1A). To assess the effect of TGF-β1 deficiency in tumor cells on the development of autochthonous mammary tumors, we monitored the mice for tumor growth. We found no differences between *Tgfb1*^f/n^ *Mmtvcre*-PyMT and their littermates *Tgfb1*^f/n^-PyMT when evaluated for tumor latency, tumor burden at the age of 20 weeks or the age at which the tumors reach the predetermined size of 2500 mm^3^ (Figure 1B and 1C).

Tumor pathogenesis has remarkable complexity that can manifest itself at more than one stage in the evolution of tumors. Thus, primary tumor size per se may not capture the full functional spectrum of a given signaling pathway on carcinogenesis. Metastatic dissemination is the primary cause of tumor-associated death in a very high proportion of cancer patients[59]. Tumor progression in the PyMT model recapitulates key features of human breast cancer including high incidence of secondary tumor development in the lung[56]. When *Tgfb1*^f/n^ *Mmtvcre*-PyMT mice and the *Tgfb1*^f/n^-PyMT cohorts with comparable tumor burden were evaluated for pulmonary metastasis, we found comparable tumor burden in the lungs (Figure 1D). These observations collectively suggest that tumor growth and metastasis in PyMT mice do not require TGF-β1 produced by tumor cells. Inactivation of T cell TGF-β1 inhibits tumor development in PyMT mice

**Figure 2:**
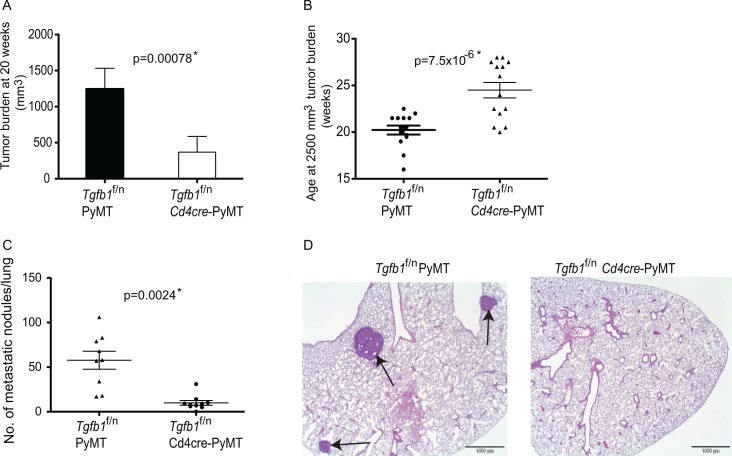
Deficiency of T cell TGF-β1 inhibits mammary tumor development (A) Total tumor burden in all mammary glands of *Tgfb1*^f/n^ *Cd4cre*-PYMT (n=14) and control Tgfb1^f/n^ PYMT (n=14) mice at 20 weeks of age. Tumor volume was calculated using the formula L x W^2^x 0.52 (where L is the longest diameter and W is the smallest diameter.) (B) The age at which *Tgfb1*^f/n^ *Cd4cre*-PYMT (n=14) and littermate control *Tgfb1*^f/n^ PYMT mice (n=14) achieved the predetermined tumor burden of 2500mm^3^. Tumor volume was calculated using the formula L x W^2^x 0.52 (where L is the longest diameter and W is the smallest diameter.) (C) Total pulmonary metastatic nodules in *Tgfb1*^f/n^ *Cd4cre*-PYMT (n=9) and control *Tgfb1*^f/n^ PYMT (n=9) mice were assessed at a tumor burden of 2500 mm^3^. The p values between the two groups of tumor burden (A) age (B) and number of metastatic nodules (C) are shown (Students t-test). * depicts statistically significant difference. (D) Histological analysis of sections of lungs of *Tgfb1*^f/n^ *Cd4cre*-PYMT and *Tgfb1*^f/n^ PYMT at a tumor burden of 2500 mm^3^. Sections of representative lungs from *Tgfb1*^f/n^ PYMT and *Tgfb1*^f/n^ *Cd4cre*-PYMT mice were stained with hematoxylin and eosin. Black arrows indicate metastatic nodules.

By virtue of the widely reported role of TGF-β1 produced by tumor cells in tumor development[44, 51-53], it was quite unexpected to find that deletion of TGF-β1 from tumor cells neither affected primary tumor growth nor tumor metastasis compared to control littermates (Figure 1B, 1C and 1D). Next, we turned out attention to the role of TGF-β1 produced by T cells in the development of PyMT tumors. In our earlier studies, we demonstrated that T cells provide the critical source of TGF-β1 that promotes immunotolerance and tumor development in the TRAMP model of prostate cancer[18]. Specifically, we demonstrated that the inhibitory effect of TGF-β1 resides in TGF-β1 produced by Ox40^+^ subpopulation of T cells that consists predominantly of activated CD4^+^ T cells and CD4^+^Foxp3^+^ T cells (Tregs)[60]. Thus, we became interested in the broader applicability of T cell produced TGF-β1 in tumor development. To investigate the function of T cell-derived TGF-β1 in mammary carcinogenesis, we crossed the *Tgfb1*^f/n^ *Cd4cre* line to PyMT mice to generate *Tgfb1*^f/n^ *Cd4cre*-PyMT mice. When evaluated for tumor growth, we found that tumor burden at 20 weeks of age was significantly lower in *Tgfb1*^f/n^ *Cd4cre*-PyMT mice compared to control *Tgfb1*^f/n^-PyMT mice (Figure 2A). In cancer patients, higher pathological grade correlates with poorer survival outcome[62]. By histopathological analysis, we found that, on average, 40% of the mammary glands in *Tgfb1*^f/n^*Cd4cre*-PyMT mice were normal whereas over 60% of the mammary glands analyzed in *Tgfb1*^f/n^-PyMT mice had histological grade III or higher (Table 1). Thus a considerable proportion of the mammary glands in *Tgfb1*^f/n^*Cd4cre*-PyMT mice were prevented from advancing to higher histological grades. In contrast In addition, the tumors in *Tgfb1*^f/n^ *Cd4cre*-PyMT mice grew at a slower rate reaching a volume of 2500 mm^3^ in approximately 25 weeks of age whereas it took *Tgfb1*^f/n^-PyMT mice only 20 weeks to reach the same tumor burden (Figure 2B). Thus, tumor latency is increased and tumor growth kinetics reduced in *Tgfb1*^f/n^ *Cd4cre*-PyMT mice compared to littermate *Tgfb1*^f/n^-PyMT mice.

**Table 1:** Pathologic scoring for stages of tumor development in the mammary glands of littermates of *Tgfb1*^f/n^ *Cd4cre*-PYMT and *Tgfb1*^f/n^ PYMT mice at 20 weeks of age The values are average percentages of the stages of 4 pairs of littermates reviewed by identifying tumor stages by area and expressing each stage as a % ± SEM The normal % represents the percentage area within the mammary gland with no visible tumor.

Stage	Normal (%)	I (%)	II (%)	III (%)	IV (%)
**Tgfb1^f/n^-PyMT**	16.5±6.8	11.6±3.7	9.3±5.8	6.9±3.9	55.6±12.3
**Tgfb1^f/n^ Cd4cre-PyMT**	39.7±26.0	20.7±9.1	22.0±8.6	9.2±4.2	8.4±16.8

The requirement for mammary tumor growth on TGF-β1 produced by T cells is consistent with our previous reports[18]. However, in the TRAMP model, it was not possible to select mice with comparable tumor burden, a parameter that has been shown to be coupled to metastatic risk[61]. We circumvented this limitation by employing B16-OVA and EL-4 metastasis assay where we found that, similar to primary tumor growth, T cell-produced TGF-β1 was required for the metastasis of these tumors[18]. Although experimental metastasis using B16-OVA and EL-4 tumors are well-established assays, they nonetheless have the important caveat of not recapitulating physiological metastasis. We found that whereas tumor development in *Tgfb1*^f/n^-PyMT mice evoked significant secondary tumor development in the lungs, pulmonary tumor metastasis was largely inhibited in *Tgfb1*^f/n^*Cd4cre*-PyMT mice that had reached similar primary tumor burden as *Tgfb1*^f/n^-PyMT mice (Figure 2C and 2D). These results collectively establish that TGF-β1 produced by T cells promotes tumor growth and progression and further facilitates secondary niche colonization in PyMT mice. DiscussionTumor growth is a complex phenomenon. In addition to the initiating oncogenic signals, tumors require additional capabilities, which along with a permissive environment, facilitate tumor cell survival, proliferation and metastasis[1]. Studies have implicated the cytokine TGF-β1 in tumor initiation and progression[1, 48, 58]. However, because diverse cell types produce TGF-β1, defining its cellular source for tumor growth has been challenging. Using a spontaneous model of mammary cancer, we demonstrated that deleting TGF-β1 from tumors did not protect mice against tumor development. However, inactivation of TGF-β1 in T cells significantly increased tumor latency and inhibited tumor growth. Additionally, we showed that T cell produced TGF-β1 is necessary for tumor progression to higher pathological grades and is further required for the development of secondary tumors in the lungs. These findings reveal T cells as the critical source of TGF-β1 that promotes mammary tumor development.

TGF-β1 has long been recognized as a cytokine that promotes tumor development and it is thought that this cytokine mediates tumor growth via its direct effect on tumors[1, 48, 58]. Indeed, mice that express a dominant negative mutant of TGF-βRII (DNR) in the prostate epithelium have accelerated tumor development of TRAMP tumors [41], supporting the function of TGF-β1 as an enforcer of cytostasis in a variety of cell types[48, 57]. Yet, to what degree physiological TGF-β signaling influences tumor development remains incompletely resolved. Studies show that another important target of TGF-β1 in tumors is antitumor immune responses[2, 39, 48, 63]. In agreement with this observation, we demonstrated recently that T cell responsiveness to TGF-β induces T cell tolerance in TRAMP mice[18]. Accordingly attenuation of TGF-β signaling in T cells protects TRAMP mice from tumor development whereas tumors develop normally in control TRAMP mice[18]. In PyMT mice in which TGF-β signaling is blocked in T cells, tumor development is similarly inhibited (our unpublished observations). While there is evidence suggesting that TGF-β sensitivity by tumors plays an important role for tumor initiation and progression, our data support a model in which the inhibitory effect of TGF-β on the immune system is more important and ultimately determines tumor growth and its blockade in T cells can override these growth advantages TGF-β signaling confers to tumors.

A crucial observation in this study is the finding that deletion of TGF-β1 from tumor cells did not have any consequences on tumor latency or progression in PyMT mice. Furthermore, we found that tumor-derived TGF-β1 is dispensable for CD4^+^ and CD8^+^ T cell differentiation in PyMT mice (data not shown). Many cell types produce TGF-β1 and because of the broad distribution of the TGF-β receptors, almost every cell type responds to this cytokine[29]. Our findings are in stark contrast to the widely held hypothesis that tumor-derived TGF-β1 promotes T cell tolerance and tumor development [2, 38, 44, 48, 52, 53, 64]. The favored mechanism is that tumors, by mechanisms largely unknown but probably involve hypoxia and/or inflammation, acquire the ability to abundantly produce TGF-β1 during their evolution which enables the tumors to progress via autocrine effects on tumors and paracrine suppression of immunosurveillance. This model derives from findings described in a study showing that overexpression of active TGF-β1 cDNA in immunogenic tumors increased their tumorigenicity and evaded CTL-mediated eradication[53]. Succeeding studies further showed improved antitumor immunity via antibody neutralization of TGF-β or tumor cell expression of soluble TGF-βRII. However, it is evident that a requirement for tumor-derived TGF-β1 in immunotolerance and tumor development cannot be deduced from these observations. Consistent with our findings, a more recent study provided evidence that shRNA-mediated knockdown of tumor-derived TGF-β1 did not inhibit tumor development in tumor transplantation studies whereas tumor cell expression of soluble TGF-βRII did[54]. Whether the development of other spontaneous tumors and, more importantly, human cancers will also prove to be independent of tumor-derived TGF-β1 as demonstrated for TRAMP [18] and PyMT tumors is open for future inquiries.

As opposed to tumor-derived TGF-β1, deletion of TGF-β1 from T cells significantly increased tumor latency and suppressed tumor development in PyMT mice. However unlike *Tgfb1*^f/n^*Cd4cre*-TRAMP mice where tumor protection was associated with frank increases in the expression of the effector molecules IFN-γ and GzmB by T cells in the tumor-draining lymph nodes and prostates[18], tumor protective immunity in *Tgfb1*^f/n^*Cd4cre*-PyMT mice was associated with moderately increased expression of these effector molecules in *Tgfb1*^f/n^*Cd4cre*-PyMT mice compared to *Tgfb1*^f/n^-PyMT mice (data not shown). The IFN-γ and the killer cell cytotoxic granule pathways are essential for the rejection of a variety of tumor types[7, 9, 10, 13-15]. However, to what extent the observed responses account for the inhibition of tumor development in these mice remains unclear. Nonetheless, these observations bring to one additional tumor in which deletion of TGF-β1 from T cells protects mice against spontaneous tumors. These findings however raise an important question: what special attributes endow TGF-β1 produced by T cells with the ability to suppress antitumor immune responses? One possible answer lies in the biological properties of TGF-β1. Because TGF-β1 is secreted as an inactive latent ligand, it requires activation catalyzed by multiple processes. One well-established *in vivo* mechanism of activation is via dendritic cell (DC)-expressed αvβ8 integrin[31]. Therefore, we speculate that in the process of priming naïve T cells in the tumor-draining lymph nodes, T cells secrete TGF-β1, which in turn regulates T cell responses following TGF-β1 activation by DCs. There are two data sets to support this speculation. First, in the TRAMP model, we previously found that tumor development triggers enhanced TGF-β-dependent Smad2 and Smad3 phosphorylation in the tumor-draining lymph nodes compared to other tissues examined including the prostate[18]. We further showed that TGF-β inhibition of T cell priming in the tumor-draining lymph nodes is contributed partly by TGF-β1 produced by T cells[18]. Second, and more recently, we found that the inhibitory effect of T cell TGF-β1 resides predominantly in activated Ox40^+^ T cells[60]. In an unrelated process, data from pharmacokinetic studies have demonstrated that active TGF-β1 has an extremely short half-life of 2-3 min[65]. This short half-life, combined with the ubiquitous expression of TGF-β receptors by tumors and cells in the tumor stroma, makes it unlikely for tumor-derived TGF-β1 to successfully diffuse to the tumor-draining lymph nodes to regulate T cell responses.

The propensity of primary tumors to undergo EMT, become invasive, to seed and colonize distant niches has important clinical implications[39, 59]. Tumor metastasis accounts for over 90% of cancer-related deaths[59]. In human breast cancer, a recent study demonstrated that TGF-β plays a key role in priming tumor cells for dissemination and adaptation to secondary locations such as the lung although the cellular source of TGF-β that mediates this process was not investigated[57]. We previously found that TGF-β1 produced by T cells is necessary for lung and peritoneal cavity colonization in experimental metastasis assays[18]. In this report, we have further extended these findings by demonstrating that pulmonary metastasis of PyMT tumors is dependent on TGF-β1 produced by T cells. In agreement with our previous observations[18], secondary tumor development in the lungs was comparable between *Tgfb1*^f/n^-PyMT and *Tgfb1*^f/n^*Mmtvcre*-PyMT mice, formally demonstrating that TGF-β1 produced by these tumors is dispensable for autochthonous tumor metastasis. These findings collectively refine and expand upon our previous findings showing that TGF-β1 produced by T cells but not tumors crucially participates in tumor development by suppressing antitumor immune responses to foster primary tumor growth and metastatic dissemination.

The elucidation of the molecular mechanisms that underlie carcinogenesis has led to rapid informed development of many mechanism-based therapies for cancer. Typically, these therapeutic agents inhibit molecular targets that regulate one or more cancer hallmark capabilities. Experimental evidence shows that because of redundancy in signaling pathways or the acquisition of new genetic or epigenetic changes, tumors, more often than not, adapt to the therapeutic agent[66]. Thus, successful eradication of cancer will require various drug combinations that target parallel signaling pathways that undergird one or more cancer hallmarks. Given the side effects associated with inhibiting a single molecular target, this goal seems so far away if not impossible.

Immunotherapy is an emerging viable alternative for cancer therapy. Recent clinical and animal model studies have demonstrated the effectiveness and durability of cancer immunotherapy[67]. The success of Ipilimumab/Yervoy, recently approved by the FDA for the treatment of metastatic melanoma is a sterling example of how the immune system can be harnessed to fight cancer. The TGF-β pathway is an important target for therapeutic intervention in cancer. Indeed, various strategies to modulate TGF-β signaling are already in different stages of clinical evaluation including neutralizing antibodies, silencing oligonucleotides and small molecule inhibitors. All these approaches lack specificity because their mechanism is based on targeting TGF-β systemically. Our findings suggest that strategies that deliver TGF-β blockade specifically to T cells alone can alleviate immunotolerance and potentially overrule the cell-intrinsic hallmarks of cancer. Such rational approaches would not only leverage antitumor immune response but also preserve the cytostatic effects of TGF-β on tumors for cancer eradication. Methods

### Mice

PyMT and mice with floxed and null alleles of *Tgfb1* gene have previously been described[56, 68]. PyMT mice were backcrossed to C57BL/6 background for 10 generations. Crossing mice with floxed and null alleles of *Tgfb1* gene (f/n) with *CD4cre* mice generated *Tgfb1*^f/n^*Cd4cre* mice having T cell-specific deletion of TGF-β1. We subsequently generated *Tgfb1*^f/n^*Cd4cre*-PyMT mice by crossing *Tgfb1*^f/n^*Cd4cre* to PyMT mice. Tumor cell-specific deletion of TGF-β1 was achieved by crossing PyMT^f/n^ mice with *Mmtvcre* mice (Jackson Laboratory, stock#003553) that had been backcrossed to C57BL/6 background for 10 generations. All mice were maintained under specific pathogen-free conditions, and animal experimentation was conducted in accordance with MSKCC institutional guidelines.

### Reverse transcription PCR

RNA was prepared from FACS-sorted tumor cells and lymph node cells with miRNeasy kit (Qiagen), and was reverse transcribed into cDNA. cDNA was amplified with the primer set for TGF-b1 gene: 5'-ATCCCACCTTTGCCGAGGGTT-3', and 5'-GCTTCCCGAATGTCTGACGTA-3'. Histopathology and pulmonary nodule enumeration

Mammary glands and lungs from sacrificed animals were fixed in 10% buffered neutral formalin and embedded in paraffin. For macroscopic pulmonary nodule analysis, lungs were fixed in Bouin's fixative and the number of metastases counted with a dissecting microscope. For histopathological analyses, 4-5 μm tissue sections were routinely stained with haematoxylin and eosin and microscopically examined for the stages of tumor development. Microscopic samples were semi-quantitatively reviewed by identifying tumor stages by area and each stage was expressed as a percentage of the tissue sample. Tumor stage classification followed the criteria as previously described[69]. Statistical analysis

Student's t test was used to calculate statistical significance for difference in a particular measurement between groups. A p value of < 0.05 was considered statistically significant.
